# Social participation patterns and associations with subsequent cognitive function in older adults with cognitive impairment: a latent class analysis

**DOI:** 10.3389/fmed.2025.1493359

**Published:** 2025-02-27

**Authors:** Xin Li, Haishan Feng, Qingling Chen

**Affiliations:** Zhongshan Hospital, Xiamen University, Xiamen, China

**Keywords:** older, cognitive impairment, social participation, latent class analysis, Chinese

## Abstract

**Background:**

Social participation (SP) has been associated with cognitive benefits among older adults; however, little is known about SP patterns and their relationships with cognitive function in cognitively impaired populations. This study aimed to identify SP patterns among cognitively impaired older individuals and evaluate their associations with subsequent cognitive function, specifically mental intactness and episodic memory.

**Methods:**

Data were drawn from the China Health and Retirement Longitudinal Study (CHARLS), including 5,673 individuals aged 60 years and older with cognitive impairment from 2015 to 2018. Latent class analysis (LCA) was used to identify SP patterns, and hierarchical linear regression analyses were conducted to examine associations between these patterns and cognitive function.

**Results:**

Four distinct SP patterns were identified: “Offline Social Participation Group” (Class 1), “Intellectual Social Participation Only Group” (Class 2), “Club and Volunteer Activities Group” (Class 3), and “Minimal Social Participation Group” (Class 4). The Class 4 comprised the majority (73%) and exhibited the poorest cognitive outcomes. Compared to the Class 4, Class 2 showed significant improvements in mental intactness (*p* < 0.001) and episodic memory (*p* = 0.022), while Class 3 demonstrated significant improvements in mental intactness (*p* = 0.032) but not episodic memory. Class 1 showed significant improvements in episodic memory (*p* = 0.023).

**Conclusion:**

This study highlights the cognitive benefits of social participation, particularly intellectual activities, for older adults with cognitive impairment. Targeted interventions promoting SP, especially intellectual activities, are essential to mitigate cognitive decline and improve cognitive resilience in vulnerable populations.

## Introduction

With the global population aging rapidly, age-related cognitive impairment has become a critical public health challenge (1). Such impairments often lead to the loss of daily functional abilities, increasing dependency and imposing significant burdens on families, healthcare systems, and society at large (2). Therefore, exploring strategies to promote healthy brain aging and identifying practical interventions are essential. This need is particularly urgent in low- and middle-income countries, where specialized medical resources are limited, and a substantial portion of the population faces cognitive impairments (3, 4).

Recent studies have increasingly explored the impact of social participation on cognitive function (5–8). Investigating social participation (SP) patterns in older adults with cognitive impairment is crucial, as this population faces specific challenges, such as cognitive barriers, social withdrawal, and physical limitations, which affect their ability to engage socially. Understanding SP patterns can inform tailored interventions that address these challenges while harnessing the protective effects of SP against further cognitive impairment (9, 10).

Social participation, defined as involvement in activities involving interaction with others in society or the community beyond the household (11), may help prevent cognitive decline through several mechanisms. First, SP can stimulate cognitive activities and strategic thinking while promoting synaptic density and neural growth (12). Second, physical activities associated with SP may improve cerebral blood flow, enhance aerobic capacity, and support brain nutrition, reducing the risk of cognitive disorders (13). Finally, SP can foster interpersonal relationships, increase social support, reduce psychological stress, and mitigate neurogenic changes contributing to cognitive decline (14).

While the positive effects of social participation on cognitive decline are widely acknowledged (15, 16), knowledge gaps remain, particularly for older adults already experiencing cognitive impairment. Due to the broad range of activities classified as social participation, consensus on which specific types benefit cognitive function most has yet to be reached. Many previous studies have focused on general relationships between variables (6, 17), often overlooking individual diversity in SP patterns, which may lead to overestimated associations or spurious correlations. Additionally, a key limitation is the failure to address heterogeneity in SP patterns among older adults with cognitive impairment.

To address these challenges, our study employs a person-centered latent class analysis (LCA) approach combined with a longitudinal design, allowing the identification of distinct SP patterns and their prospective effects on cognitive function. Most existing studies have only examined direct associations between SP and cognitive function, often including limited covariates such as demographics, while neglecting socioeconomic and health-related variables. Considering these covariates is crucial for developing targeted interventions.

Latent class analysis (18) categorizes individuals into unobserved subgroups (latent classes) with similar participation patterns, facilitating the modeling of cognitive heterogeneity. This method is particularly valuable for identifying unique SP patterns within diverse populations, such as older adults with cognitive impairment. By classifying these patterns, LCA helps identify subgroups that may benefit most from targeted interventions while accounting for the diverse ways SP interacts with cognitive function. Leveraging LCA enables the use of non-discrete manifest variables to explain underlying relationships and classify SP patterns of older adults with cognitive impairment into shared categories.

Therefore, the objective of this study is to identify SP patterns among older adults with cognitive impairment in China and determine variations in influencing factors across patterns. Furthermore, through longitudinal analysis, we aim to explore the relationship between these SP patterns and cognitive function outcomes over 3 years. Our findings aim to provide substantial evidence supporting the protective role of social participation in older adults with cognitive impairment.

## Methods

### Participants

This study utilized data from two survey waves of the China Health and Retirement Longitudinal Study (CHARLS): specifically, the 2015 (wave 3) and 2018 (wave 4) datasets (19). Data from 2015 served as the baseline, while data from 2018 focused on assessing cognitive function using Mini-Mental State Examination (MMSE) scores, which included both mental intactness and episodic memory components. The CHARLS project, managed by the China Center for Economic Research at Peking University, this study included adults aged 45 and older, along with their spouses. CHARLS gathered data on demographic details, socio-economic conditions, and health-related behaviors through face-to-face interviews conducted in participants’ homes. Participants were selected using a multi-stage probability-proportional-to-size sampling method. This method involved random sampling based on geographic regions, urban districts or rural counties, and per capita GDP. CHARLS covered 28 provinces in China, including residents from 150 counties. Our study specifically focused on individuals aged 60 and older who exhibited cognitive impairment, as determined by MMSE scores. Initially, we included 18,016 participants from the 2015 survey. However, some participants were excluded for various reasons, resulting in a final cohort of 5,673 individuals (Supplementary Figure S1). Cognitive impairment was defined using MMSE scores, following a previous study (20). Specifically, individuals with less than elementary education and an MMSE score of 17 or lower were classified as having cognitive impairment. Those with elementary education and an MMSE score of 20 or lower, as well as participants with middle school education or higher and an MMSE score of 24 or lower, were also categorized as cognitively impaired.

### Assessment of cognitive function

This study employed the MMSE for the evaluation of cognitive function. The MMSE, as used in CHARLS, consists of two segments: mental intactness and episodic memory, both of which have received validation in Chinese demographics (21). The component of mental intactness is derived from facets of the Telephone Interview of Cognitive Status, and the scores, ranging from 0 to 11, equate to the total number of accurate responses. The episodic memory segment is predicated on immediate and delayed word recall assessments, with scores corresponding to the sum of correctly reiterated words and spanning from 0 to 20 (22). In this study, we employed the 2018 mental intactness scores and episodic memory scores as separate outcome measures to assess the cognitive function of the older population with cognitive decline. In this paper, the Cronbach’s α coefficients for the years 2015, and 2018 were 0.80, and 0.82, respectively.

### Assessment of social participation

There is one question in CHARLS regarding social participation: “Have you engaged in any of these social activities in the past month?.” Participants were deemed to have “social participation” if they were involved in any one of the subsequent activities: (1) interacting with neighbors or friends socially, (2) participating in games like ma-jong, chess, or cards, or joining a community club, (3) providing unpaid assistance to non-cohabiting family, friends, or neighbors, (4) attending a sports, social, or other club, (5) engaging in community-related organizations, (6) contributing to voluntary or charitable work, (7) caring for a non-cohabiting sick or disabled adult, (8) enrolling in an educational or training course, and (9) using the Internet. Participants who did not partake in any of these activities were categorized as “not having social participation.” Those who confirmed involvement in the 9th activity were labeled as having “online participation (ONP).” Participants engaging in at least one of the first eight activities were classified as having “offline participation (OFP).” Based on previous literature (23, 24), OFP was further categorized into four specific types in this study. Questions (4) and (5) were identified as club activities (CLA), while questions (2) and (8) were designated as intellectual activities (INA). Question (1) was categorized as simple interpersonal activities (SIA), and questions (3, 6) and (7) were grouped under volunteer activities (VOA). Hence, there are a total of five categories of social participation in this study: ONP, CLA, INA, SIA, and VOA.

### Covariates

Based on previous research (25), we included demographic and health-related factors from the 2015 baseline as covariates in our analysis. The demographic covariates included age, gender, dwelling place, marital status, and educational level. Health-related covariates included baseline cognitive function level, current smoking and alcohol consumption habits, physical activity, self-rated health scores, depressive symptoms, hearing impairment, distance vision impairment, near vision impairment, activities of daily living (ADL) scores, and reported pain. Specific details about the measurements and methods used to assign these variables are provided in the Supplementary files. It is noteworthy that the presence of depressive symptoms was evaluated using a 10-item scale from the Center for Epidemiologic Studies Depression Scale (CES-D-10), with a threshold of 10 or higher indicating a positive result (26). Previous research (27) showed that the CES-D-10 demonstrated excellent internal consistency (Cronbach α = 0.69–0.89), sensitivity (71.4–84.6%), and specificity (72.6–95%) for depression screening.

### Statistical analysis

Continuous variables in this study were assessed for normal distribution using quantile-quantile plots, which indicated general adherence to a normal distribution. Means and standard deviations described continuous variables, while categorical variables were presented as frequencies and percentages. Latent class analysis (LCA) was conducted to identify distinct subgroups based on the five social participation categories. All analyses were performed using Mplus software (version 8.3; Muthen & Muthen, Los Angeles, California). Fit indices, including the Bayesian information criterion (BIC), Akaike information criterion (AIC), Sample Size-Adjusted BIC (aBIC), and entropy test, were used to determine the optimal number of profiles (28). Model comparison employed the Lo–Mendell–Rubin likelihood ratio test (LMR) and bootstrapped likelihood ratio test (BLRT). Typically, the best-fitting model was assessed based on lower Bayesian information criterion, Akaike information criterion, and sample size-adjusted BIC values. Well-fitting models yielded entropy values of 0.80 or higher, and the Lo–Mendell–Rubin likelihood ratio test and bootstrapped likelihood ratio test were used to compare alternate models. The best-fitting model was chosen based on these fit indices and model interpretability. Once the optimal latent subgroups were identified, sociodemographic, health-related variables, and cognitive function outcomes were assessed using SPSS (version 24.0; IBM Corp, Armonk, New York). Categorical variable comparisons utilized the chi-square test, while continuous variables were analyzed with analysis of variance.

The association between latent classes and subsequent cognitive function in older individuals with cognitive decline was examined through hierarchical linear regression analysis. Sociodemographic and health-related characteristics significantly associated with cognitive function in univariate analyses were included as control variables in Model 1. The independent variables (distinct latent classes) were added in Model 2. Dummy variables were generated for both distinct latent classes and levels of education within the hierarchical regression analysis. All tests were two-tailed, with statistical significance set at a *p* value of less than 0.05.

## Results

### Characteristics of the participants

The characteristics of the participants are detailed in Table 1. Among the total of 5,673 participants, 3,122 (55.0%) were male, while 2,551 (45.0%) were female. Predominance was observed among participants aged between 65 and 70, accounting for 68.8%. Nearly half of the participants (49.7%) reported possessing a primary school education. The majority of participants were married (83.2%), and only 26.6% of them resided in urban areas. Furthermore, 31.1% of participants reported current smoking habits, and 21.2% reported current drinking habits. Physical activity was reported by 43.0% of participants. Depressive symptoms were indicated by 33.3% of participants. Additionally, 30.8% of participants reported experiencing pain. Hearing impairment was noted by 15.7% of participants, while 23.4% reported distance vision impairment. Near vision impairment was reported by 19.1% of participants. The mean scores for ADL and self-rated health were 5.07 ± 3.34 and 3.98 ± 0.89, respectively.

**Table 1:** Differences in characteristics among the latent classes.

Characteristics	Class 1: Offline social participation group (*n* = 215)	Class 2: Intellectual social participation only group (*n* = 902)	Class 3: Club and volunteer activities participation group (*n* = 415)	Class 4: Minimal social participation group (*n* = 4,141)	*χ* ^2^ */F*	*p* value
Age (year), *n* (%)					4.579	0.205
60 ≤ Age < 70	150 (69.8)	643 (71.3)	294 (70.8)	2,818 (68.1)		
Age ≥ 70	65 (30.2)	259 (28.7)	121 (29.2)	1,323 (31.9)		
Gender, *n* (%)					55.550	<0.001
Male	143 (66.5)	579 (64.2)	201 (48.4)	2,199 (53.1)		
Female	72 (33.5)	323 (35.8)	214 (51.6)	1,942 (46.9)		
Marital status, *n* (%)					4.035	0.258
Married	184 (85.6)	765 (84.8)	350 (84.3)	3,420 (82.6)		
Single/other	31 (14.4)	137 (15.2)	65 (15.7)	721 (17.4)		
Dwelling place, *n* (%)					258.857	<0.001
Urban area	74 (34.4)	313 (34.7)	227 (54.7)	893 (21.6)		
Rural area	141 (65.6)	589 (65.3)	188 (45.3)	3,248 (78.4)		
Education level, *n* (%)					231.406	<0.001
No formal education	26 (12.1)	117 (13.0)	45 (10.9)	951 (23.0)		
Primary school	82 (38.1)	433 (48.0)	157 (37.8)	2,145 (51.8)		
Middle school and above	107 (49.8)	352 (39.0)	213 (51.3)	1,045 (25.2)		
Current smoker, *n* (%)					73.000	<0.001
No	119 (55.3)	544 (60.3)	327 (78.8)	2,917 (70.4)		
Yes	96 (44.7)	358 (39.7)	88 (21.2)	1,224 (29.6)		
Current drinker, *n* (%)					16.578	<0.001
No	138 (64.2)	617 (68.4)	318 (76.6)	2,995 (72.3)		
Yes	77 (35.8)	285 (31.6)	97 (23.4)	1,146 (27.7)		
Physical activity, *n* (%)					5.021	0.170
No	115 (53.5)	530 (58.8)	220 (53.0)	2,371 (57.3)		
Yes	100 (46.5)	372 (41.2)	195 (47.0)	1770 (42.7)		
Depressive symptoms, *n* (%)					82.465	<0.001
No	161 (74.9)	684 (75.8)	319 (76.9)	2,617 (63.2)		
Yes	54 (25.1)	218 (24.2)	96 (23.1)	1,524 (36.8)		
Pain, *n* (%)					45.089	<0.001
No	156 (72.6)	700 (77.6)	303 (73.0)	2,766 (66.8)		
Yes	59 (27.4)	202 (22.4)	112 (27.0)	1,375 (33.2)		
Hearing impaired, *n* (%)					34.796	<0.001
No	186 (86.5)	811 (89.9)	364 (87.7)	3,421 (82.6)		
Yes	29 (13.5)	91 (10.1)	51 (12.3)	720 (17.4)		
Distance vision impaired, *n* (%)					30.790	<0.001
No	175 (81.4)	735 (81.5)	342 (82.4)	3,094 (74.7)		
Yes	40 (18.6)	167 (18.5)	73 (17.6)	1,047 (25.3)		
Near vision impaired, *n* (%)					27.324	<0.001
No	190 (88.4)	759 (84.1)	356 (85.8)	3,283 (79.3)		
Yes	25 (11.6)	143 (15.9)	59 (14.2)	858 (20.7)		
ADL scores, mean (SD)	4.49 (3.21)	4.47 (3.29)	4.43 (3.26)	5.29 (3.34)	23.337	<0.001
Self-rated health scores, mean (SD)	3.82 (0.92)	3.92 (0.86)	3.80 (0.93)	4.02 (0.89)	12.083	<0.001
Baseline mental intactness scores	7.15 (3.03)	6.62 (3.35)	7.41 (3.18)	5.43 (3.40)	77.861	<0.001
Baseline episodic memory scores	8.13 (2.57)	8.06 (2.62)	8.31 (2.57)	6.75 (3.14)	80.143	<0.001

ADL, activities of daily living.

### Latent class analysis of social participation

**Figure 1:**
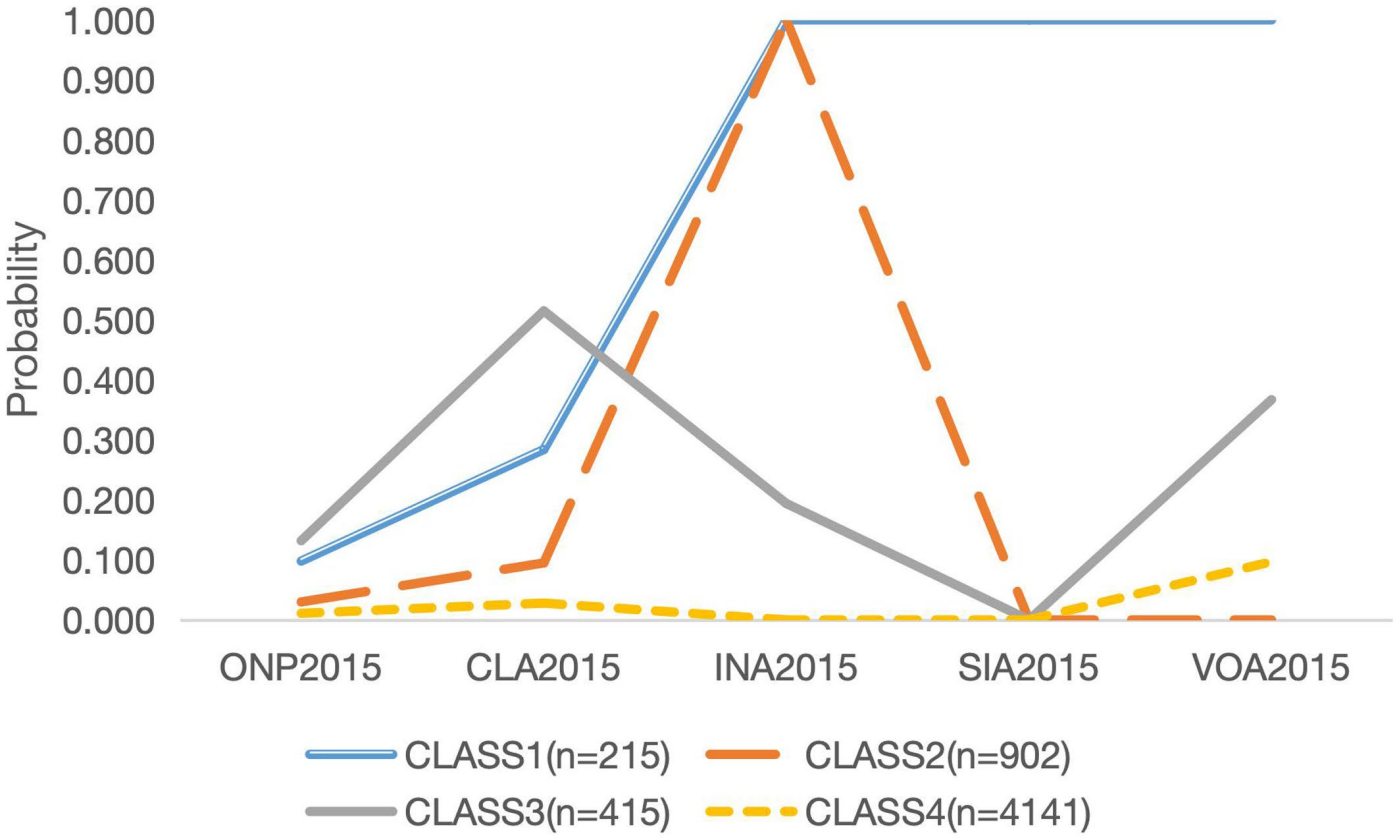
Category probability plot of the 4 latent class analysis in the study (*n* = 5,673). ONP, online participation; CLA, club activities; INA, intellectual activities; SIA, interpersonal activities; VOA, volunteer activities.

Table 2 presents the fit indices for latent class models ranging from 1 to 5. The AIC, BIC, and aBIC, utilized to assess goodness of fit, exhibited a continuous decrease from class 1 to class 4, followed by an increase in class 5. Only the entropy values of the two-class and four-class models fell within the range of 0.8 to 1. The two-class and five-class models were excluded due to elevated AIC, BIC, and aBIC values. The three-class model was excluded based on entropy considerations. Furthermore, the 4-class model displayed a more sensible distribution of case numbers and class probabilities. Although one of the groups within the 4-class classification exhibited a probability less than 5%, its absolute count surpassed 50. Given the clinical significance associated with its presence, this categorization was considered valid. In conclusion, the 4-class model emerged as the most fitting. Figure 1 illustrates the distribution of three potential classes characterized by varying levels of participation.

**Table 2:** Latent class model fit indices.

Class	LL	AIC	BIC	aBIC	Entropy	LRT, *p*	BLRT, *p*	Class probability
One-class	−8674.798	17359.597	17392.814	17376.926	-			-
Two-class	−7829.761	15681.522	15754.600	15719.646	0.999	0.000	0.000	0.038/0.962
Three-class	−7740.063	15514.127	15627.066	15573.045	1.000	0.000	0.000	0.856/0.045/0.100
Four-class^a^	−7690.216	15426.431	15579.231	15506.144	0.850	0.000	0.000	0.038/0.159/0.073/0.730
Five-class	−7688.868	15435.736	15628.397	15536.243	0.798	0.592	0.500	0.038/0.014/0.159/0.771/0.019

AIC, Akaike information criteria; BIC, Bayesian information criterion; BLRT, bootstrap likelihood ratio test; BLRT, bootstrap likelihood ratio test; LL, log-likelihood.

a Four-class was selected as the final latent class model.

### Characteristics of classes

Table 1 illustrates distinct characteristics of the individuals within each group. Employing outcomes from the LCA analysis, we conducted a comparison of social participation across four classes. The characteristics of each group were analyzed from the chi-square test results or variance analysis results. With the exception of marital status, physical activity, and comorbidity, other characteristics exhibited noteworthy differences among the four classes (*p* < 0.05).

Class 1, labeled as “Offline Social Participation Group,” accounted for 3.8% (215/5,673) of the total cohort. This group exhibited a strong propensity to actively engage in a variety of social activities, particularly in INA (Intellectual Activities), SIA (Simple Interpersonal Activities), and VOA (Volunteer Activities). Additionally, within this class, there was a higher proportion of males, as well as a lower prevalence of current smokers and current drinkers. Class 2, referred to as “Intellectual Social Participation Only Group,” constituted 15.9% (902/5,673) of the sample. Older people with cognitive impairment in Class 2 exhibited the highest likelihood of participating solely in INA (Intellectual Activities), with a relatively narrow focus on this type of participation, showing minimal involvement in other forms of social participation. In comparison to the other categories, this class had the lowest proportion of individuals with hearing impairment. Class 3, designated as “Club and Volunteer Activities Participation Group,” accounted for 7.3% (415/5,673) of the sample. The defining characteristic of this class was the high likelihood of participation in CLA (Club Activities) and VOA (Volunteer Activities), while involvement in SIA (Simple Interpersonal Activities) and ONP (Online Participation) was almost negligible. Moreover, in comparison to the other three classes, Class 3 encompassed the highest number of female participants, individuals with at least a middle school education, non-current smokers, individuals involved in physical activities, and those without symptoms of depression or distance vision impairment. Additionally, both ADL and self-rated health scores were the lowest in this class. Class 4, referred to as “Minimal Social Participation Group,” constituted the largest segment of the sample at 73.0% (4,141/5,673). This category displayed minimal levels of social involvement within the study. A significant majority of older adults fell into this class, with a notable proportion residing in rural areas. Additionally, a lower proportion of individuals had attained education beyond middle school, with a prevalent representation of those with no formal education or primary school education. Furthermore, this group demonstrated a higher prevalence of individuals exhibiting depressive symptoms, reporting pain, experiencing hearing impairment, distance vision impairment, and near vision impairment. Correspondingly, their ADL scores and self-rated health scores were comparatively higher than those of the other groups.

### Hierarchical linear regression

To better estimate the association between social participation and subsequent cognitive function in older adults with cognitive impairment, we set the social participation’s latent classes to dummy variables and used the mental intactness scores and episodic memory scores as the dependent variables for hierarchical linear regression analyses. As shown in Table 3, after adjusting for age, gender, marital status, education level, dwelling place, current smoking status, current drinking status, self-rated health scores, physical activity, depressive symptoms, ADL scores, pain, hearing impairment, distance vision impairment, near vision impairment, and baseline mental intactness scores (Model 2), significant associations were observed between distinct subgroups and mental intactness scores. Specifically, Class 2 compared to Class 4 (*β* = 0.037, *p* < 0.001) and Class 3 compared to Class 4 (*β* = 0.023, *p* = 0.032) demonstrated significant positive associations with mental intactness scores. In contrast, Class 1 compared to Class 4 (*β* = 0.020, *p* = 0.054) showed a marginal association with mental intactness scores, but the difference was not statistically significant. The overall model was significant (*F* = 187.422, *p* < 0.001), with subgroup classifications (Classes 2 and 3), along with age, gender, marital status, education level, dwelling place, ADL scores, hearing impairment, and baseline mental intactness scores, collectively accounting for 39.9% of the variance in mental intactness scores (adjusted *R^2^* = 0.397).

**Table 3:** Hierarchical linear regression analysis for the subsequent mental intactness scores.

Variables	Model 1				Model 2a			
	*B*	SE	*β*	*p*	*B*	SE	*β*	*p*
Age	−0.604	0.078	−0.084	<0.001	−0.602	0.077	−0.084	<0.001
Gender	−0.272	0.088	−0.041	0.002	−0.275	0.088	−0.041	0.002
Marital status	−0.117	0.048	−0.026	0.014	−0.119	0.048	−0.027	0.013
Education level = Primary school	0.916	0.097	0.138	<0.001	0.910	0.096	0.137	<0.001
Education level = Junior high school and above	1.931	0.116	0.267	<0.001	1.900	0.117	0.263	<0.001
Dwelling place	−0.390	0.084	−0.052	<0.001	−0.351	0.084	−0.047	<0.001
Current smoker	−0.043	0.029	−0.018	0.129	−0.050	0.029	−0.021	0.083
Current drinker	0.004	0.028	0.002	0.885	0.005	0.028	0.002	0.851
Self-rated health scores	0.081	0.044	0.022	0.065	0.081	0.044	0.022	0.064
Physical activity	0.132	0.069	0.020	0.058	0.132	0.069	0.020	0.057
Depressive symptoms	−0.130	0.083	−0.018	0.119	−0.111	0.083	−0.016	0.180
ADL scores	−0.029	0.012	−0.029	0.017	−0.027	0.012	−0.027	0.023
Pain	−0.051	0.086	−0.007	0.590	−0.051	0.086	−0.007	0.555
Hearing impaired	−0.266	0.100	−0.029	−0.255	−0.442	0.100	−0.028	0.011
Distance vision impaired	−0.020	0.091	−0.003	0.827	−0.019	0.090	−0.002	0.834
Near vision impaired	−0.076	0.095	−0.009	0.419	−0.070	0.095	−0.008	0.461
Baseline mental intactness scores	0.476	0.013	0.440	<0.001	0.471	0.013	0.435	<0.001
Latent class								
Class 4: Minimal social participation group (Ref.)								
Class 1: Offline social participation group					0.350	0.182	0.020	0.054
Class 2: Intellectual social participation only group					0.338	0.097	0.037	<0.001
Class 3: Club and volunteer activities participation group					0.293	0.137	0.023	0.032
*F*	218.979^a^				187.422^a^			
*R^2^*	0.397				0.399			
Adjusted *R^2^*	0.395				0.397			
Δ*R^2^*					0.002^a^			

B, unstandardized coefficient; ref., reference; SE, standard error; β, standardized coefficient.

a *p value less than 0.001.*

Furthermore, to provide a more detailed comparison of the effects of different social participation patterns on subsequent mental intactness, we conducted additional analyses using Class 1 (Supplementary Table S2), Class 2 (Supplementary Table S3), and Class 3 (Supplementary Table S4) as reference groups in turn. The findings indicated that, compared to older adults with cognitive impairment who exhibited minimal social participation (Class 4), all other SP patterns (except the Class 1) generally had a positive impact on subsequent mental intactness. However, the differences between the various SP patterns were not statistically significant. (*p* values were all more than 0.05).

In our second analysis (Table 4), after adjusting for age, gender, marital status, education level, dwelling place, self-rated health scores, physical activity, depressive symptoms, ADL scores, pain, hearing impairment, distance vision impairment, near vision impairment, and baseline episodic memory scores (Model 2), distinct subgroups showed significant associations with episodic memory scores (Class 1 vs. Class 4: *β* = 0.026, *p* = 0.023; Class 2 vs. Class 4: *β* = 0.027, *p* = 0.022). However, the comparison between Class 3 and Class 4 (*β* = 0.015, *p* = 0.199) did not reach statistical significance. The overall model was significant (*F* = 102.343, *p* < 0.001), with the subgroups (Classes 1 and 2) along with age, marital status, education level, dwelling place, current smoking status, ADL scores, hearing impairment, and baseline episodic memory scores collectively accounting for 26.6% of the variance in episodic memory scores (adjusted *R^2^* = 0.263).

**Table 4:** Hierarchical linear regression analysis for the subsequent episodic memory scores.

Variables	Model 1				Model 2a			
	*B*	SE	*β*	*p*	*B*	SE	*β*	*p*
Age	−0.570	0.101	−0.068	<0.001	−0.572	0.101	−0.068	<0.001
Gender	0.094	0.113	0.012	0.404	0.098	0.113	0.013	0.387
Marital status	−0.248	0.062	−0.048	<0.001	−0.249	0.062	−0.048	<0.001
Education level = Primary school	1.144	0.122	0.147	<0.001	1.136	0.122	0.146	<0.001
Education level = Junior high school and above	2.392	0.147	0.283	<0.001	2.359	0.147	0.279	<0.001
Dwelling place	−0.519	0.108	−0.059	<0.001	−0.487	0.109	−0.055	<0.001
Current smoker	−0.073	0.037	−0.026	0.048	−0.080	0.037	−0.029	0.030
Current drinker	−0.048	0.036	−0.017	0.184	−0.047	0.036	−0.016	0.191
Self-rated health scores	0.013	0.057	0.003	0.816	0.014	0.057	0.003	0.798
Physical activity	0.055	0.090	0.007	0.537	0.055	0.090	0.007	0.537
Depressive symptoms	−0.033	0.107	−0.004	0.757	−0.016	0.107	−0.002	0.878
ADL scores	−0.049	0.015	−0.042	0.002	−0.048	0.015	−0.041	0.002
Pain	−0.245	0.111	−0.029	0.027	−0.245	0.111	−0.029	0.027
Hearing impaired	−0.144	0.129	−0.013	0.265	−0.136	0.129	−0.013	0.292
Distance vision impaired	0.078	0.117	0.009	0.505	0.078	0.117	0.009	0.506
Near vision impaired	0.033	0.122	0.003	0.786	0.043	0.122	0.004	0.726
Baseline episodic memory scores	0.363	0.014	0.322	<0.001	0.359	0.015	0.317	<0.001
Latent class								
Class 4: Minimal social participation group (Ref.)								
Class 1: Offline social participation group					0.535	0.236	0.026	0.023
Class 2: Intellectual social participation only group					0.286	0.125	0.027	0.022
Class 3: Club and volunteer activities participation group					0.227	0.177	0.015	0.199
*F*	119.673^a^				102.343^a^			
*R^2^*	0.265				0.266			
Adjusted *R^2^*	0.262				0.263			
Δ*R^2^*					0.001^b^			

B, unstandardized coefficient; ref., reference; SE, standard error; β, standardized coefficient.

a *p value less than 0.001.*

b *p value less than 0.05.*

To further compare the effects of different social participation patterns on subsequent episodic memory, we performed additional analyses by alternately using Class 1 (Supplementary Table S5), Class 2 (Supplementary Table S6), and Class 3 (Supplementary Table S7) as reference groups. The results showed that, while all other social participation patterns (except the Class 3) generally had a positive influence on subsequent episodic memory compared to the minimal social participation group (Class 4), the differences between these patterns were relatively minor, with all *p* values exceeding 0.05.

## Discussion

In this study, we investigated various patterns of social participation and their associations with subsequent cognitive function in older adults with cognitive impairment, specifically examining mental intactness scores and episodic memory scores. Using latent class analysis (LCA), we identified four distinct social participation subgroups: “Offline Social Participation Group” (Class 1), “Intellectual Social Participation Only Group” (Class 2), “Club and Volunteer Activities Participation Group” (Class 3), and “Minimal Social Participation Group” (Class 4). Notably, the majority of participants (73%) were classified into Class 4, indicating a considerable prevalence of low social engagement. Importantly, our results revealed that all other forms of social participation, regardless of type, were generally associated with a positive impact on subsequent cognitive function compared to minimal participation. This emphasizes the cognitive benefits of social engagement. Among the identified groups, Class 2 (intellectual participation only) demonstrated significant positive associations with both mental intactness and episodic memory scores, highlighting the unique value of intellectual activities for older adults with cognitive impairment.

Our research findings emphasize that a significant portion of the older population experiencing cognitive impairment exhibits limited social engagement, particularly in intellectual and interpersonal activities. Amano et al. (29) investigated social engagement patterns among MCI patients in the United States and identified three distinct categories: exclusive informal social engagement, a combination of formal and informal social engagement, and low levels of social engagement. Notably, the latter group accounted for 32.8% of the cohort studied. In our study, this proportion more than doubled, potentially due to the inclusion of both MCI and dementia patients in our sample. As cognitive decline progresses, social engagement levels tend to decrease further. Furthermore, when considering the context of China as a developing nation (8) and the predominantly rural composition of our study population, the extent of social engagement is even more limited. Luo et al. (30) examined social engagement among elderly individuals in western Chinese communities using the Participation and Autonomy Questionnaire (IPA), their analysis revealed three tiers of social engagement: low, moderate, and high. The moderate engagement profile emerged as the predominant category, encompassing 55.1% of participants. It is important to note that their methodology differed from ours, as it did not emphasize cognitive health, which could explain the higher participation rates among non-cognitively impaired individuals. Moreover, their survey employed a door-to-door approach within a local residential area, thereby introducing inherent biases. Hence, drawing an exhaustive comparison between our study and previous research is intricate due to methodological disparities. Nevertheless, it is evident that the degree of social engagement within the cognitively impaired elderly demographic significantly lags behind that of their cognitively normal counterparts, particularly across diverse forms of participatory activities.

In our study, participants classified in the “minimal social participation group” exhibited lower scores in both the mental intactness and episodic memory scores. This finding reinforces previous researches (8, 31) and underscores the importance of enhancing subsequent cognitive function in older individuals who have already experienced cognitive impairment. Additionally, as cognitive data were collected 3 years later, this further confirms that low social participation can serve as a predictive factor for cognitive decline, emphasizing the need for targeted interventions for vulnerable populations. Our hierarchical regression analyses confirmed that distinct social participation patterns are associated with cognitive outcomes. Compared to the “Minimal Social Participation Group” (Class 4), individuals in Class 2 and Class 3 exhibited significant cognitive benefits, particularly in mental intactness scores. “Mental intactness scores” serve as a comprehensive measure encompassing various cognitive aspects, including attention, memory, and executive function. Social participation can influence cognitive performance in these domains, as social interactions engage complex cognitive processes such as attention, working memory, task switching, and memory. Thus, considering social participation as a form of “brain training” is reasonable, as it helps preserve neural networks and buffers against age-related cognitive decline. Episodic memory was also enhanced among participants in Class 1 (offline participation) and Class 2 (intellectual participation), suggesting that engaging in social interactions and intellectual tasks fosters cognitive resilience. Social participation may act as a form of “cognitive training” stimulating processes such as working memory, attention, and executive function, which are integral to overall cognitive performance (32, 33). “Episodic memory scores” primarily assess an individual’s ability to recall personal experiences and events. Social participation might influence an individual’s capacity for event recall since social interactions often entail interactions with others, shared experiences, and conversations—all of which can stimulate an individual’s episodic memory.

Although differences between specific social participation patterns were relatively minor, our results consistently demonstrated that even minimal engagement confers cognitive benefits. For instance, participants in the “Club and Volunteer Activities Group” (Class 3) also showed significant associations with mental intactness scores, albeit to a lesser degree than intellectual participation (Class 2). Episodic memory scores, which primarily assess event recall, were similarly improved in groups engaging in offline and intellectual participation. These results support the notion that social interactions and shared experiences inherent in such activities stimulate episodic memory (34). However, participants in Class 3 did not achieve statistical significance in episodic memory outcomes, suggesting that intellectual participation offers unique cognitive advantages compared to other forms of engagement.

In China, as population aging accelerates, the number of older adults experiencing cognitive impairment is becoming increasingly substantial (35). Therefore, delaying cognitive decline in this group is critically important, as it could significantly alleviate China’s economic and social burden. Social participation, as a relatively economically feasible intervention approach (36), can yield beneficial effects for the elderly population that has already undergone cognitive decline.

### Limitations

This study has several limitations. First, the observational design limits our ability to establish causal relationships between social participation and its influencing factors. However, the longitudinal nature of the study allows us to explore prospective associations between social participation and cognitive function. Second, due to the feasibility constraints in data collection, we were unable to assess the quality of social participation, which may limit our understanding of how different types of social participation contribute to mitigating cognitive decline. Third, our study focused primarily on categorizing the types of social participation without considering the frequency of engagement, which could provide additional insights into its impact. Fourth, cognitive function was measured using the MMSE scale rather than clinical diagnoses, which might influence the strength of the observed associations between social engagement and subsequent cognitive function. Furthermore, the MMSE may not accurately reflect complex cognitive processes such as episodic memory. Future studies should consider incorporating additional assessment tools for a more comprehensive evaluation.

## Conclusion

This study highlights the cognitive advantages of social participation for older adults with cognitive impairment. Engaging in any form of social activity provides certain benefits for subsequent cognitive function, with intellectual activities showing particularly positive effects on both mental intactness and episodic memory. Promoting interventions aimed at increasing social participation among older adults with cognitive impairment could be an effective strategy for mitigating cognitive decline and enhancing quality of life. Efforts should be made to actively encourage their participation in any form of social activity while providing opportunities for intellectual social participation whenever possible.

## Data Availability

The datasets presented in this study can be found in online repositories. The names of the repository/repositories and accession number(s) can be found below: CHARLS database.
