# Supplementary material for: Social participation patterns and associations with subsequent cognitive function in older adults with cognitive impairment: a latent class analysis
Source: Front Med (Lausanne). 2025 Feb 27;12:1493359. doi: 10.3389/fmed.2025.1493359 (PMC11903710; doi:10.3389/fmed.2025.1493359)
Supplement: Supplementary file 1 [file Data_Sheet_1.PDF]

Supplementary Files

Table S1 List of study variables considered in the present study among older adults in the 2015 and 2018 CHARLS study

Variable	Definition
Age	60-65, 65+
Sex	Female, Male
Marital status	Married/with partner, Other
Education level	Illiterate, Primary school, Middle school and above
Dwelling place	Rural, Urban
Current smoker	Yes, No
Current drinker	Yes, No
Comorbidity	Whether they had comorbidities or not (No, Yes)
ADL score	Numerical (the higher the score, the worse of the mobile ability)
Self-rated health score	Numerical (the higher the score, the worse of the health)
Depressive symptoms	Whether there are depressive symptoms (No, Yes)[a] , measured by CES-D
Physical inactivity	Whether they had physical activity or not (No, Yes)
Pain	Whether they had pain or not (No, Yes)
Hearing impaired	Whether they had hearing impaired or not (No, Yes)
Distance vision impaired	Whether they had distance vision impaired or not (No, Yes)
Near vision impaired	Whether they had near vision impaired or not (No, Yes)

Note: CHARLS, China Health and Retirement Longitudinal Study; ADL, activities of daily life; CES-D, Center for Epidemiologic Studies Depression Scale

[a]Presence of depressive symptoms is indicated by a CES-D score greater than or equal to 10

Table S2 Hierarchical linear regression analysis for the subsequent mental intactness scores (Model 2b)

	Model 2b			
Latent class	*B*	SE	β	*P*
Class 1: Offline social participation group (Ref.)				
Class 2: Intellectual social participation only group	-0.013	0.196	-0.001	0.948
Class 3: Club and volunteer activities participation group	-0.058	0.218	-0.005	0.792
Class 4: Minimal social participation group	-0.350	0.182	-0.047	0.054
F	187.422[a]			
R^2	0.399			
Adjusted R^2	0.397			
ΔR^2	0.002[a]			

Note: Adjusted for Model 1's variables; *B*, unstandardized coefficient; ref., reference; SE, standard error; β, standardized coefficient; [a] *P* value less than 0.001.

Table S3 Hierarchical linear regression analysis for the subsequent mental intactness scores (Model 2c)

	Model 2c			
Latent class	*B*	SE	β	*P*
Class 2: Intellectual social participation only group (Ref.)				
Class 1: Offline social participation group	0.013	0.196	0.001	0.948
Class 3: Club and volunteer activities participation group	-0.045	0.155	-0.004	0.773
Class 4: Minimal social participation group	-0.338	0.097	-0.045	<0.001
F	187.422[a]			
R^2	0.399			
Adjusted R^2	0.397			
ΔR^2	0.002[a]			

Note: Adjusted for Model 1's variables; *B*, unstandardized coefficient; ref., reference; SE, standard error; β, standardized coefficient; [a] *P* value less than 0.001.

Table S4 Hierarchical linear regression analysis for the subsequent mental intactness scores (Model 2d)

	Model 2d			
Latent class	*B*	SE	β	*P*
Class 3: Club and volunteer activities participation group (Ref.)				
Class 1: Offline social participation group	0.058	0.218	0.003	0.792
Class 2: Intellectual social participation only group	0.045	0.155	0.005	0.773
Class 4: Minimal social participation group	-0.293	0.137	-0.039	0.032
F	187.422[a]			
R^2	0.399			
Adjusted R^2	0.397			
ΔR^2	0.002[a]			

Note: Adjusted for Model 1's variables; *B*, unstandardized coefficient; ref., reference; SE, standard error; β, standardized coefficient; [a] *P* value less than 0.001.

Table S5 Hierarchical linear regression analysis for the subsequent episodic memory scores (Model 2b)

	Model 2b			
Latent class	*B*	SE	β	*P*
Class 1: Offline social participation group (Ref.)				
Class 2: Intellectual social participation group	-0.249	0.254	-0.023	0.326
Class 3: Low interpersonal activities participation group	-0.308	0.282	-0.021	0.275
Class 4: Minimal social participation group	-0.535	0.236	-0.061	0.023
F	102.343[a]			
R^2	0.266			
Adjusted R^2	0.263			
ΔR^2	0.001[b]			

Note: Adjusted for Model 1's variables; *B*, unstandardized coefficient; ref., reference; SE, standard error; β, standardized coefficient; [a] *P* value less than 0.001; [b] *P* value less than 0.05.

Table S6 Hierarchical linear regression analysis for the subsequent episodic memory scores (Model 2c)

	Model 2c			
Latent class	*B*	SE	β	*P*
Class 2: Intellectual social participation only group (Ref.)				
Class 1: Offline social participation group	0.249	0.254	0.012	0.982
Class 3: Club and volunteer activities participation group	-0.059	0.200	-0.004	0.789
Class 4: Minimal social participation group	-0.286	0.125	-0.033	0.022
F	102.343[a]			
R^2	0.266			
Adjusted R^2	0.263			
ΔR^2	0.001[b]			

Note: Adjusted for Model 1's variables; *B*, unstandardized coefficient; ref., reference; SE, standard error; β, standardized coefficient; [a] *P* value less than 0.001; [b] *P* value less than 0.05.

Table S7 Hierarchical linear regression analysis for the subsequent episodic memory scores (Model 2d)

	Model 2d			
Latent class	*B*	SE	β	*P*
Class 3: Club and volunteer activities participation group (Ref.)				
Class 1: Offline social participation group	0.308	0.282	0.015	0.275
Class 2: Intellectual social participation only group	0.059	0.200	0.006	0.769
Class 4: Minimal social participation group	-0.227	0.177	-0.026	0.199
F	102.343[a]			
R^2	0.266			
Adjusted R^2	0.263			
ΔR^2	0.001[b]			

Note: Adjusted for Model 1's variables; *B*, unstandardized coefficient; ref., reference; SE, standard error; β, standardized coefficient; [a] *P* value less than 0.001; [b] *P* value less than 0.05.

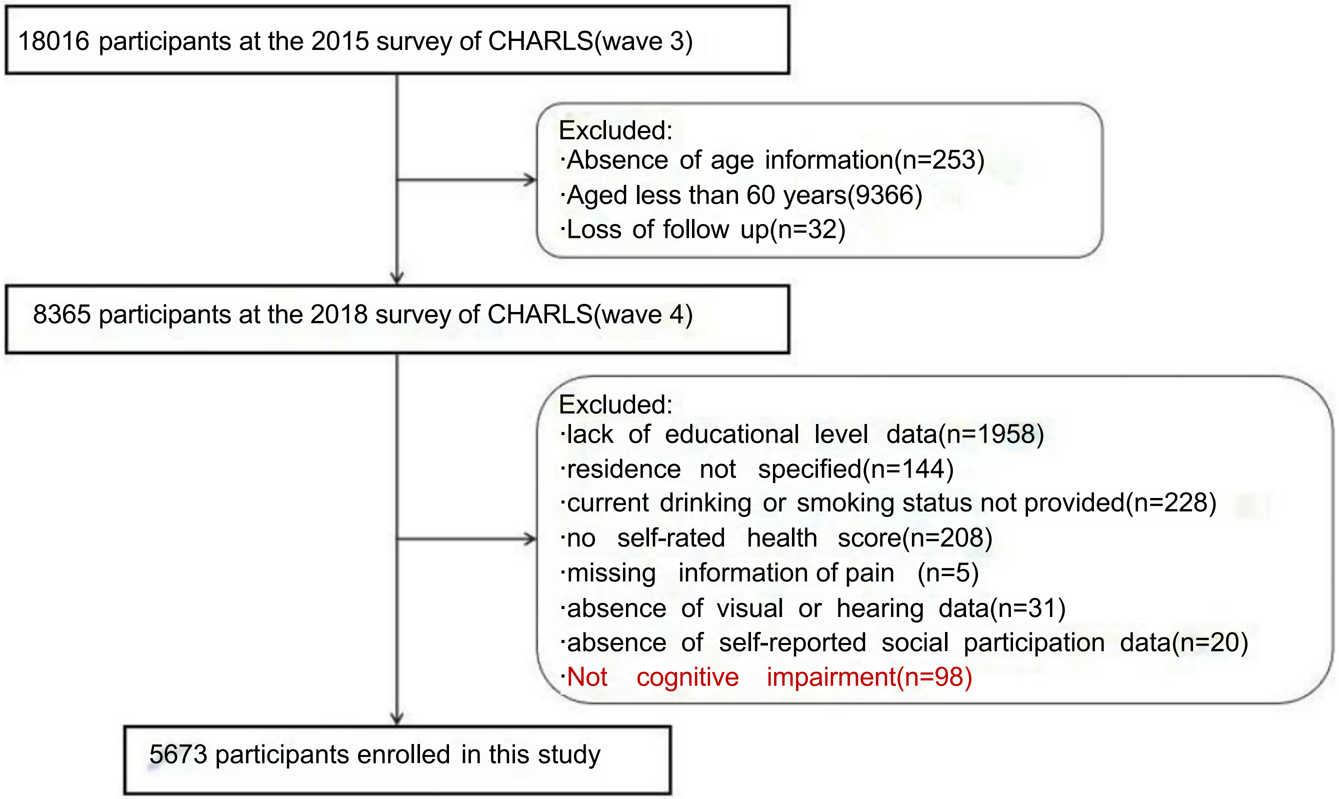

Figure S1 Flowchart of this study.
